# A Portable Electromagnetic System Based on mm-Wave Radars and GNSS-RTK Solutions for 3D Scanning of Large Material Piles

**DOI:** 10.3390/s21030757

**Published:** 2021-01-23

**Authors:** Humberto Fernández Álvarez, Guillermo Álvarez-Narciandi, María García-Fernández, Jaime Laviada, Yuri Álvarez López, Fernando Las-Heras Andrés

**Affiliations:** Area of Signal Theory and Communications, University of Oviedo, 33203 Gijón, Spain; alvareznguillermo@uniovi.es (G.Á.-N.); garciafmaria@uniovi.es (M.G.-F.); laviadajaime@uniovi.es (J.L.); alvarezyuri@uniovi.es (Y.Á.L.); flasheras@uniovi.es (F.L.-H.A.)

**Keywords:** mm-wave radars, GNSS-RTK positioning, wireless technology, electromagnetic scanning, point cloud

## Abstract

In this paper, a portable three-dimensional (3D) scanning system for the accurate characterization of large raw material (e.g., cereal grain, coal, etc.) stockpiles is presented. The system comprises an array of high resolution millimeter-wave radars and a cm-level accuracy positioning system to accurately characterize large stockpiles by means of a high-resolution 3D map, making it suitable for automation purposes. A control unit manages the data received by the sensors, which are sent to a computer system for processing. As a proof of concept, the entire sensor system is evaluated in a real environment for electromagnetically scan a scaled stockpile of coal, used in the industry for handling raw materials. In addition, a highly efficient processing adaptive algorithm that may reconstruct the scanned structure in real-time has been introduced, enabling continuous dynamic updating of the information. Results are compared with those from a photogrammetry-like technique, revealing an excellent agreement.

## 1. Introduction

Industry 4.0 entails the full interconnection between systems and devices and the employment of massive amounts of data to make predictive decision models. These new challenges are of vital importance to the development of the fourth industrial revolution and settle the basis to the full automation of industrial processes. The machine to machine (M2M) communication is a key challenge to be faced in this context, being crucial for avoiding collisions among machines or with surrounding obstacles and hence, it has attracted the interest of many researchers [1,2]. It should be noticed that there is a large amount of contributions that deal with collision avoidance among vehicles [3,4,5,6]. These systems are usually based on detecting objects or structures in a certain perimeter and emitting signals to the machine, so that it can dodge them. However, this bare detection is not enough to fully automatize certain processes, nor to provide an instantaneous decision based on the gathered data. Consequently, additional information is sometimes required, such as the shape and/or volume of certain structures or areas, whose constant update is usually needed, as it may be subjected to continuous changes. Moreover, industrial environments are usually exposed to hazardous conditions, which have to also be taken into account.

Accordingly, the searching of non-invasive techniques to retrieve the actual topography of different areas has been widely pursued during the recent years, as it has a great number of applications not only for industrial solutions, but also for analyzing dense forested terrains [7] or ever-changing river surfaces [8].

The information regarding space availability and stored material in certain areas such as docks, industrial plants and/or warehouses is of vital importance for planning and managing processes. An interesting case of use is the analysis of the available stockpile of raw materials in the mining, agriculture, construction and energy industries. The information about these stockpiles is crucial for adjusting different processes such as buffering, blending, stacking, reclaiming and transporting procedures, as well as for achieving a homogeneous stockpile, which would be useful for optimizing the storage space.

The material from these stockpiles is placed by stackers from above and it is withdrawn by means of reclaimers. Reclaimers have only two degrees of freedom (longitudinal position and angle of incline), so if they are not commanded to a place with enough material they are very inefficient, resulting in a low extraction rate with the subsequent cost. For this reason, profiling stockpiles with robust sensors is very relevant as it enables to optimize the performance of the overall system. Currently, the longitudinal position and angle of incline of the reclaimer is chosen manually, requiring workers and being prone to error.

Mathematical and geometrical models have been recently developed for estimating the stockpile shape [9,10]. However, they take certain assumptions or consider ideal geometries that prevent accurately predicting the real stockpile topography [11]. An out-of-date or erroneous information about the current state of the stockpile can lead to overfilling or empty spaces (rat-holing), which may cause damage to the equipment [12] and/or an inaccurate managing of the available material, both causing long production downtimes. Moreover, the continuous stacking and reclaiming processes conducted at irregular intervals may lead to unpredictable shapes of the stockpile and prevent the fully automation of the processes. Therefore, the continuous dynamic monitoring of the stockpile is crucial for avoiding the aforementioned issues and optimizing the production costs.

The methods employed to experimentally determine the shape of stockpile raw materials have evolved during the last years, starting from the well-known Yo-Yo technique, which consisted of tying a weight to a rope for determining the stockpile height, to the deployment of different systems based on ultrasonic, radar and laser technologies [13].

There are several factors that define the most suitable technology for analyzing stockpile raw materials: material type and characteristics, environmental conditions, budget, maintenance, ease of use and installation, accuracy, external requirements (such as electrical power supply) and safety. Considering the aforementioned factors, it can be concluded that the most appropriate technology for determining the geometrical properties of the stockpiles, relies on non-invasive time-of-flight sensors [12].

Ultrasonic sensors, such as acoustic solid scanners, with wavelengths ranging from 1000 m to 100 m, have fallen into disuse as the measurements are affected by machinery noises, nearby structures, temperature, smoke and wind, as well as by other particles in the air. Moreover, they offer a slow updating rate (long latency) and low coverage [14]. Until the last few years, infrared sensors (with wavelengths ranging from 100 μm to 1 μm) have been the preferred devices for scanning stockpiles. Then, the topographic micro pulse light detection and ranging (LiDAR) technology, which provides a surface map, became of great importance for industrial companies, since it is currently moving towards the full automation of processes [11,12,13,14,15,16]. However, this technology is not suitable for areas with dense dust, smoke or gas, as the emitted signals suffer a high diffraction on these particles and it also requires permanent cleaning and maintenance [14]. In addition, the LiDAR technology may be affected by the ambient light and hence, brightness or darkness has a great impact on the detected light, which varies depending on the material surface colour (darker colour materials provide lower reflectivity), affecting the ability of the sensor to determine distances accurately [12,16].

Up to now radar technology has been underestimated, primarily due to its high manufacturing costs. However, the reduction of production costs, due to the mass-production of mm-wave radars, makes it a promising alternative to attain high resolution scanners. This technology is more suitable to work under harsh conditions, as it is robust against dust as well as independent of light or atmospheric conditions, in contrast to ultrasonic and infrared technologies. Moreover, it requires minimal maintenance, which is vital as the sensors are usually installed in emplacements with difficult access [14].

Geo-referencing the radar measurements during the scanning process is essential for post-processing the retrieved data and reconstructing the stockpile shape. In this context, encoders as well as Global Navigation Satellite System (GNSS) solutions have been adopted [15,16,17,18,19]. However, the former one, which is the most widely used in the literature, usually provides a relatively low resolution for imaging purposes, as it is designed to provide a coarse estimation of the position and its information is not always prepared to be read from external systems. Moreover, it usually entails moving the sensor through fixed paths (normally rails). Regarding the GNSS, it is very flexible, but the raw positioning accuracy is in the order of several tenths of centimeters.

Aiming at developing a versatile and portable solution, the scanner movement should not be restricted to a predefined path and, hence, a precise technique for geo-referencing the radar measurements is required. Consequently, Real-Time Kinematic (RTK) solutions, which are based on differential GNSS measurements, are ideal for determining the sensors position, as they provide cm-level accuracy position data [20]. Indeed, this geo-referring accuracy is in agreement with the degree of precision provided by the radars.

There are several commercial solutions, provided by companies such as Indurad [14] and ION [21], which use different sensors to scan, model and reconstruct stockpiles. However, these companies employ strategically positioned radars, minimizing the amount of sensors, but at the expense of a precise measurement, above all when the target is at grazing angles. Moreover, the companies do not unveil much information about the employed sensors, scanning processes and post-processing techniques, as they develop profitable *ad-hoc* solutions, which are highly customized according to the client specifications, as mentioned in [11]. Therefore, these *ad-hoc* solutions usually result in high costs (in the order of tens of thousands of euros [12]) and external maintenance.

Other works deal with simulated [17] or extremely small scaled laboratory experiments [11,12,17,18,22,23]. Hence, they did not consider real measured data, which is affected by more complex features such as noise, weather conditions and reflectivity from surrounding elements. Indeed, the measured data in a real environment can be formed by an unstructured point cloud which can be noisy, sparse and incomplete [11] and several methods have to be used to discard unwanted data, such as segmentation, noise filters and/or boundary detector techniques.

In addition, the multiple issues that usually appear during the installation and adjustment of the final system are not taken into account in these simple and limited-scope experiments.

In this paper, a robust and highly precise radar-based system to continuously scan large areas and reconstruct their topography, also suitable for managing and planning processes, is presented. Contrary to most systems described in literature, such as LiDAR, radar technology is employed, taking advantage of its performance under hazardous environmental conditions (e.g., dust, moisture) or in the absence of light. In addition, a highly precise positioning system, with an accuracy in the order of that of the radar subsystem, is deployed, allowing the merger of the point clouds of all the radars acquired at different positions, enhancing the accuracy of the resulting topography of the stockpile and enabling the scanning through irregular paths. Geo-referred radar measurements are processed, resulting in a dynamic reconstruction of the scanned structure in real-time and, hence, allowing the system to make instantaneous decisions. Consequently, the proposed system also targets improving the automation level of stockpile-related industry processes. As a proof of concept, the entire sensor system has been tested in a simulated environment for scanning a stockpile-alike model, as well as in a realistic environment for scanning a scaled coal stockpile.

## 2. Materials and Methods 

### 2.1. Three-Dimensional (3D) Profiling of Stockpiles 

**Figure 1:**
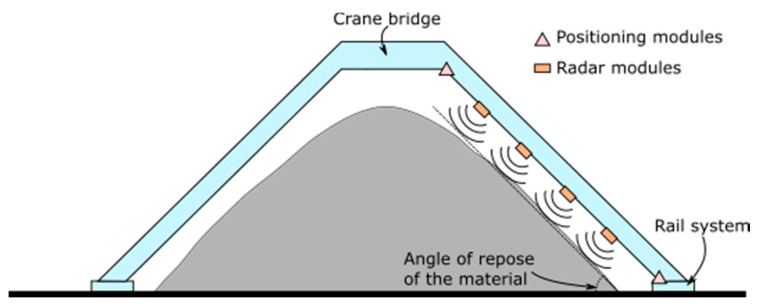
Side-view of the proposed system embedded into the crane-bridge of a stockpile management system.

In order to determine the profile of stockpiles, the sensor fusion scheme shown in Figure 1 is proposed. In this setup, several radar sensors are placed along the crane bridge that is part of the stockpile management system, and which can be moved arbitrarily through a rail system. In contrast to conventional systems, the proposed setup takes advantage of this movement to provide a high resolution three-dimension image of the stockpile with centimeter accuracy. In addition, positioning modules are used to track the position of the radars. Position and radar data are broadcasted to a laptop in order to compose the stockpile image. Thus, three subsystems are considered: (i) radar subsystem, (ii) positioning subsystem and (iii) communication and control subsystem.

In this setup, radar modules are in charge of detecting the range to the target and, therefore, they can be considered as the core sensors of the setup. In conventional setups, two options are typically used. In the first one, radars are placed on fixed positions in order to monitor the stockpile from above. Nonetheless, this kind of setup has several drawbacks. First, the view angle of each radar is limited as the stockpile or other mechanical elements could block the signal, preventing imaging some areas. As a consequence, several radars must be strategically placed in order to provide full coverage. These positions must be optimized for each stockpile facility, entailing a higher development time and increasing the cost of the solution. Moreover, the distance between the radars and the stockpile is larger in this setup, and hence the image resolution worsens [24]. The second option places one or two radars over the stockpile, typically in the top of the mobile crane bridge. This type of setup is very effective to calculate the height of the stockpile, but it is not able to provide images with grazing angles.

In the proposed solution, multistatic radar modules are placed along the crane bridge, which is typically designed with an angle close to the angle of repose of the materials. This disposal allows to achieve improved image resolutions (as the radars are usually closer to the stockpile) and avoids losing information at grazing angles. Additionally, the use of multistatic radars enables calculating a point cloud of scatterers by means of synthetic aperture radar [24]. The distance between the radars is chosen so that the overall coverage covers the entire stockpile. It is relevant to note that the distance from the stockpile to the radar modules has an impact in the point cloud resolution, as it is inversely proportional to such a distance [24].

Regarding the positioning subsystem, it is in charge of measuring the positions, so that the point clouds taken from different arbitrary positions can be merged. Since mm-wave radars can provide a resolution in the order of a few centimeters, it is important to use a positioning system with a similar accuracy, since the final stockpile resolution is dominated by the worst case. For this reason, a GNSS-RTK system is used because its accuracy is in the order of 1 cm.

Regarding the number of positioning modules, since the radars are expected to lay along a virtual line, two positioning modules are enough in order to track this line. These tracking modules should be as separated as possible in order to minimize potential alignment errors.

It is interesting to observe that radar data could be merged to create a synthetic aperture radar from arbitrary positions [25,26]. This kind of processing is very powerful as it enables very high resolution images such as the one used for people screening [27]. However, this kind of data fusion requires a spatial data sampling much smaller than the working wavelength (less than 1 cm) [28] and, consequently, it is discarded in this work.

**Figure 2:**
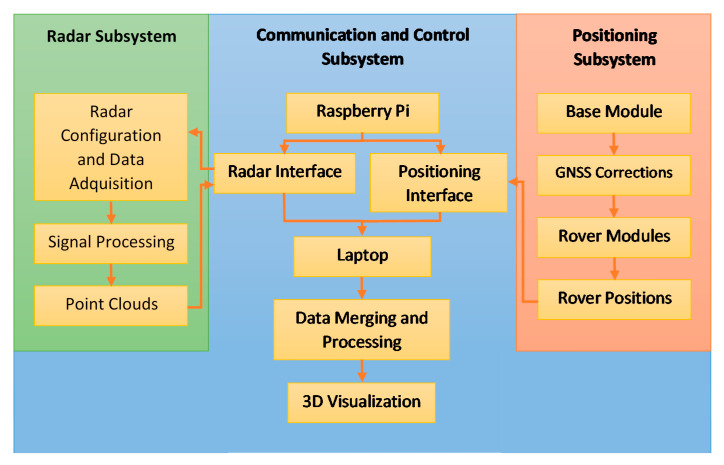
Workflow of the proposed system.

The last subsystem is designed to perform communication and control duties, using a control unit attached to the crane bridge. This subsystem is in charge of controlling the data flow among the subsystems, synchronizing the data retrieved from the radar and positioning subsystems and sending the data to a processing unit, which is basically a standard computer. A workflow of the proposed system and the aforementioned interconnection between the subsystems is presented in Figure 2.

The retrieved information from the radars and rover modules is merged on the laptop for processing and visualization purposes (as it is indicated in Figure 2). However, the point clouds provided by the radars at each measurement position are defined according to the local coordinate system (LCS) of each radar, which is neither fixed (as the radars will be moved during the scan process) nor common for all radars. Therefore, these point clouds should be manipulated to refer them to a global coordinate system (GCS), which is fixed and common to all the radars and the whole system, forming a unified point cloud. For this purpose, several coordinate system transformations, which are explained in Appendix A, are performed.

### 2.2. Components

**Figure 3:**
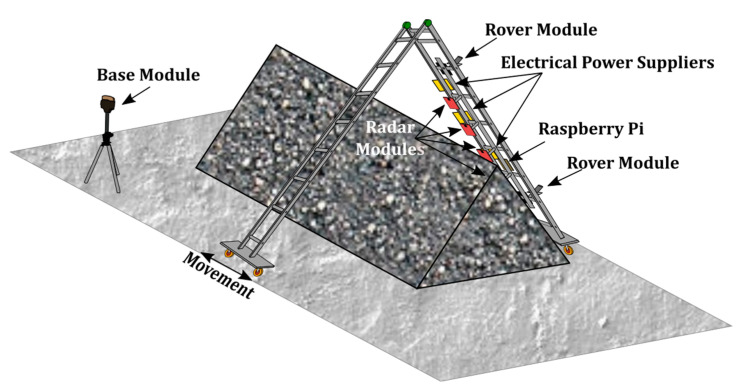
Scheme of the proposed system.

In the demonstrator used in this paper (see Figure 3), two platforms with wheels are built to accommodate a foldable ladder mimicking a piece of the crane bridge. The radar modules are fixed using *ad-hoc* 3D printed structures and the control unit consists of a laptop.

After analyzing the commercial off-the-shelf sensors, the *IWR1443* radar modules, operating in the frequency band from 76 to 81 GHz and manufactured by Texas Instruments [29] were selected for several reasons: integration of the radiofrequency elements on the board, easily control through a USB to UART interface and maximum range between 150 to 200 m (much larger than the maximum typical ranges in the order of 30–40 m commonly required [12,30]). In addition, they can be configured to provide not only the detected objects using a point cloud representation, but also the intensity of each point, which will be useful for spatial filtering unwanted echoes.

Each module comprises three transmitting and four receiving channels, allowing us to create a virtual array of twelve receiving channels and hence, improving the angular resolution. Each transmitting and receiving channel is composed of an array of three series fed patches with a 3dB-beamwidth of 28° × 56°.

The radar modules are directly connected to the control unit using USB to micro USB cables (which is the radar interface in Figure 3) and they are configured to provide a point cloud as their output data. The configuration of the radar parameters has to be conducted taking into account the deploying environment. Indeed, employing a proper configuration, it is possible to filter some spurious and undesired points, mainly caused by different factors: double reflections happening close to the radars, channel coupling or any other unwanted reflections. An *ad-hoc* GUI application running on the control unit, which sends configuration commands to the radars, was developed to configure the radars in the field. The main adjustable configuration commands are: the constant false alarm rate (CFAR), direct current (DC) and static clutter removal and peak grouping (which groups points by range and/or velocity).

Among the available GNSS-RTKs on the market, the ones manufactured by Emlid were chosen, because at the time they were acquired they offered one of the best performance-price ratio. A *Reach RS2* module [31] is intended to act as a base station, sending correction data to two *Reach M2* modules [32] that will work as rovers and will be placed on the ladder, providing a 7 to 14 mm positioning accuracy.

The GNSS-RTK communication with the control unit is based on a client-server architecture and a text protocol for acquiring latitude, longitude and height information. A Wi-Fi network is deployed to enable interchanging information between the GNSS-RTKs modules among them and with the control unit (positioning interface in Figure 3).

A portable dual band router TP-Link M7450 is used to establish the wireless communications between the subsystems, using the standard 802.11b/g/n and allowing to manage the control unit remotely through a laptop. For the proposed application, a Raspberry Pi 4 is used as the control unit of the system. However, it should be highlighted that the software implemented to manage and receive information from the sensors using the Raspberry Pi 4, can be adapted to any other control unit device, such as a programmable logic controller (PLC).

Finally, the commercial software Matlab is chosen to run the code in charge of processing the data and reconstructing and rendering the surface on the laptop.

## 3. Results

Two measurement set-ups have been considered. In the first one, the stockpile of material is simulated using air bubble aluminum laminations supported by adjustable photography background supports. This set-up, hereinafter referred to stockpile-alike model, has been deployed in the outdoors of the research group laboratory, and has been primarily devoted to analyzing and debugging the system performance, as well as for calibrating the sensors. It has also allowed us to adjust the data processing technique for reconstructing the structure.

The second set-up, which will be called a real environment, is composed of a scaled stockpile of coal deposited on the Gijón seaport.

### 3.1. Stockpile-alike Environment

**Figure 4:**
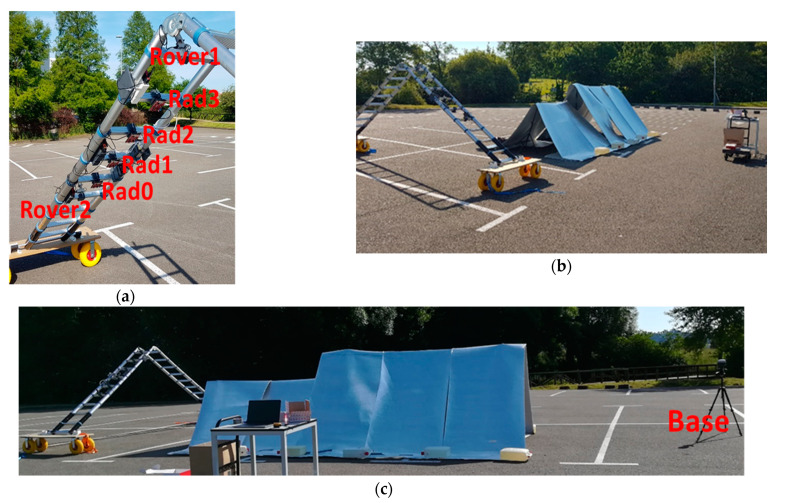
(**a**) Scheme of the sensors arrangement on the ladder; (**b**) lateral and (**c**) front views of the stockpile-alike model.

This section presents the measurement results obtained with the stockpile-alike environment. Once the required programs to control the sensors and fetch the measured data have been developed, the sensors arrangement on the ladder was set. After several tests, it was found that the best configuration for the radars and the rover modules on the ladder corresponds to the one presented in Figure 4a, where the rover modules are placed on the top and bottom of the ladder, being the radars on the middle. Each sensor is placed in the middle of each rung, being separated by 30 cm from each other, and no empty rung is left among the sensors. Taking into account that the distance from the ladder to the material is in the order of 70 cm, this setup provides a proper coverage of the scanned structure with an adequate sensors signal footprint, avoiding uncovered areas.

The stockpile-alike is disposed in two differentiating heights, as illustrated in Figure 4b,c, placed at 1.2 m and 1.5 m height, being the longitudinal dimensions of each section of 2 m and 3.4 m, respectively. Before starting the measurements, a calibration of the system should be performed to determine the relative position of the radars with respect to the rover modules. This is crucial to map the point cloud provided by each radar in its LCS to the defined GCS.

The measurements are conducted in continuous mode, setting the system to take an acquisition every half a second. Aiming at controlling the data acquisition process, several software tools have been developed, such as the continuous checking of the correct sensor operation (including the saturation level of the radar receiving chain or any communication interruption) and the monitoring of the GNSS-RTK positioning accuracy (mainly reduced due to weak signal to noise ratio reception or high dilution precision rates).

The measurements are carried out by longitudinally moving the system along the stockpile-alike model. The radars are configured with a CFAR threshold of 5 dB, so that they provide a dense point cloud. This is useful for not missing any information from the scanned structure, but it contains a large amount of undesired points (mainly from the floor and multi reflection paths). The latter is partially reduced by grouping the points by range.

The point cloud retrieved from each radar at each measurement instant is manipulated to refer it to the GCS (following the process described on Appendix A). It is worth mentioning that though the structure is designed to be linearly moved along an almost straight direction, it may suffer certain deviations that can also be corrected using the method described in the Appendix A. 

**Figure 5:**
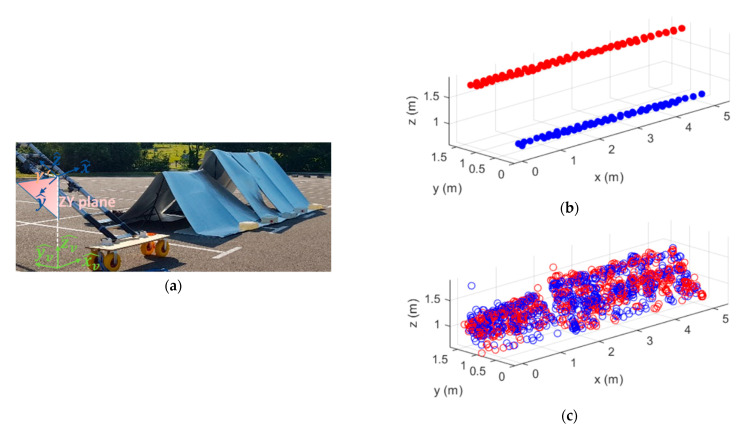
(**a**) Scanning area; (**b**) rover modules positions and (**c**) point cloud retrieved from a round trip scanning path.

In Figure 5a, the scanning area is presented. The positions of the GNSS-RTK rover modules at each measurement instant are depicted in Figure 5b (rover1 and rover2 dots in red and blue, respectively). Moreover, the retrieved filtered point cloud corresponding to a round trip scanning path, i.e., moving the system from the beginning of the scanned structure to the end and back to the beginning (Figure 5c), is also shown. The point cloud in blue corresponds to the one retrieved during the first part of the scanning acquisition (moving the system from the beginning to the end of the scanned structure, along the $\hat{x_{v}}$ direction), whereas the one in red corresponds to the data obtained during the second part of the acquisition (moving the system from the end to the beginning of the scanned structure, along the $-\hat{x_{v}}$ direction). From the point cloud results, it can be noticed a clear overlap of both point clouds (blue and red), indicating the robustness of the proposed system and method (on Appendix A) for fusing the information provided by the radars and rover modules.

Besides a proper configuration of the radars, several filters have to be employed to remove spurious and unwanted points. Therefore, a spatial filter is used to remove those points whose signal level lies below a certain threshold level. Moreover, a linear regression is applied for discarding other noisy points, coming from unwanted reflections (see Appendix A). This filtering procedure allows us to highly automatize the removal of unwanted points, even when the spatial filter is not properly adjusted. As it can be seen from Figure 5c, just a few points of the cloud are caused by spurious reflections and, therefore, it can be concluded that the filtering processes proposed are efficient. Moreover, the few unwanted remaining points lie in dispersed areas, far away from the ones caused by the scanned structure and hence, they do not result in confusing information.

**Figure 6:**
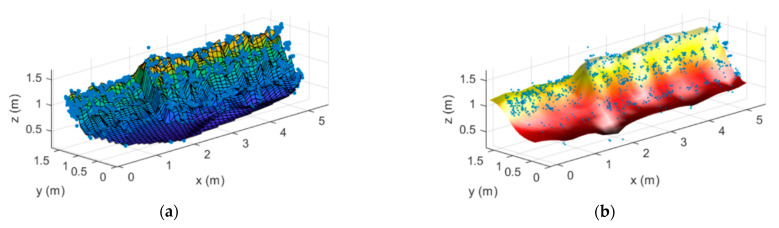
Surface reconstruction of the stockpile-alike model following (**a**) *proc1* and (**b**) *proc2* procedures.

There are several techniques for reconstructing the surface of the structure from a point cloud. In this paper, although conventional Delaunay triangulation, used in [33], has been evaluated, additional *ad-hoc* techniques have also been investigated. The first one is based on a piecewise cubic interpolation and a surface reconstruction using fitting functions to adjust the surface to the retrieved point cloud. The results are presented in Figure 6a and this procedure is called *proc1*.The second technique involves a smoothing, triangle linear interpolation and the use of the spring model, which allows us to accurately connect the points on the cloud [34]. The reconstructed surface, after applying this technique, is presented in Figure 6b and this procedure is called *proc2.* From both reconstructions, the shape and the height variations of the stockpile-alike model are clearly observed, with the height of the first and second sections of the stockpile respectively being 1.25 m and 1.53 m and their longitudinal lengths being 2 m and 3.4 m, which closely fit the true dimensions of the stockpile-alike model, as shown in Table 1. The slight discrepancies may be due to calibration errors and/or inaccuracies when acquiring the stockpile-alike model dimensions.

### 3.2. Real Environment

**Figure 7:**
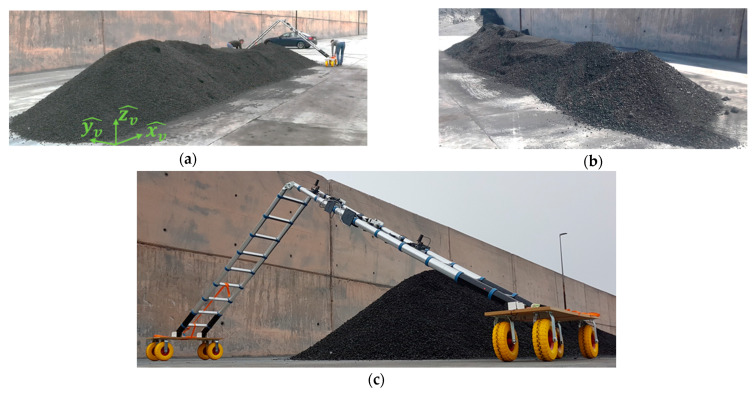
(**a**,**b**) Scaled stockpile from two different perspectives; (**c**) the ladder with the sensors prior to conducting the stockpile scanning.

Once the system was adjusted and a precise reconstruction of the stockpile-alike model was achieved, it was tested in a more realistic environment. For this purpose, a real stockpile of coal was deployed. However, in contrast to real stockpiles with heights in the order of tenths of meters, the considered model was scaled, being equivalent to a local measurement of a complete stockpile. The considered scaled-stockpile has three clear sections with different heights (see Figure 7). The first section, with a maximum height of 1.58 m, is followed by an almost flat middle area and then, a final section, slightly higher than the previous one, completes the stockpile.

The scanning procedure is identical to the one described for the stockpile-alike model. As the real stockpile has similar height dimensions as the previous stockpile-alike model and the distance from the radars to the stockpile is similar, the sensors on the ladder are placed at the same positions.

Although the stockpile was located close to a high wall, as can be seen in Figure 7, the rover modules could receive a strong enough signal from the satellites to solve their position in their fix mode and hence, a high positioning accuracy was provided.

Regarding the radars configuration, the optimum CFAR threshold has been proved to be between 5 and 13 dB, so that a dense point cloud can be obtained and no information from the scanned structure is missed. 

The data processing technique and the filtering applied to the point cloud are identical to the ones employed for the stockpile-alike model (see Appendix A for further information about data processing). Once again, a visualization coordinate system has to be defined, which is represented in Figure 7a. 

**Figure 8:**
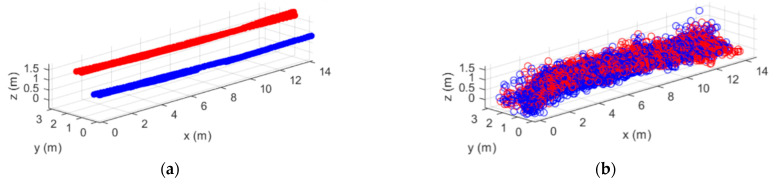
(**a**) Rover modules positions and (**b**) point cloud retrieved from a round trip scanning path.

In Figure 8a, the rover modules positions at each measurement instant are depicted (red and blue dots, respectively for rover1 and rover2). The retrieved point cloud corresponding to a round trip scanning path is shown in Figure 8b. Once again, the points corresponding to each movement direction are represented with a different color, with both point clouds being similar and again demonstrating the robustness of the proposed method for referring the point cloud to the defined GCS.

**Figure 9:**
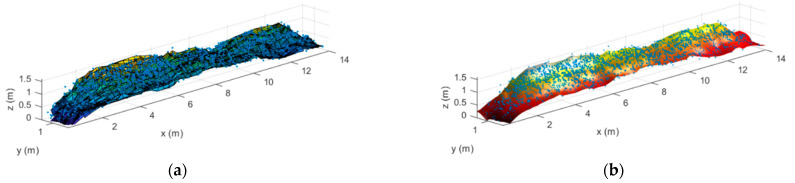
Surface reconstruction of the real stockpile following (**a**) *proc1* and (**b**) *proc2* procedures.

Then, the surface is reconstructed using both *proc1* and *proc2* and the results are shown in Figure 9. From this reconstruction, the three sections of the stockpile are clearly distinguishable, having the first one a height of 1.52 m (almost the same as the actual one, 1.58 m). The reconstructed stockpile length (14.2 m) is slightly smaller than the length of the true stockpile, as there is certain information of the stockpile base that is lost, due to the lack of an additional radar on the bottom of the ladder. However, the latter is not critical, as stockpiles are commonly uniform on its base and, consequently, no relevant information is missing. 

### 3.3. Complete Reconstruction of the Real Stockpile

Once the real stockpile has been properly scanned and its lateral surface accurately reconstructed, a complete model of the stockpile may be desired. For estimating such model, the stockpile should be scanned from both sides. As the proposed system is a prototype, including the sensors that are only on a single side, the ladder was flipped so that same sensors are used for conducting the scanning at both sides. Therefore, a round trip scanning for each side of the stockpile can be performed to fully model the stockpile. 

**Figure 10:**
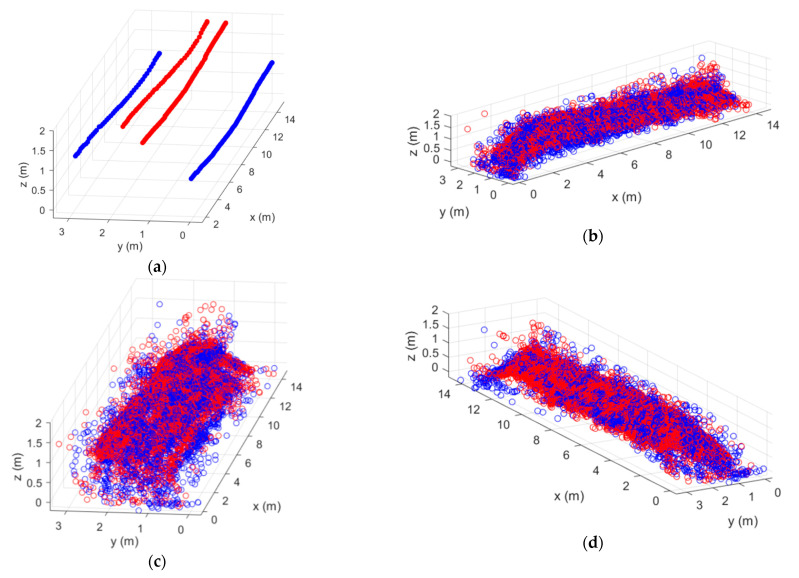
(**a**) Rover modules positions and (**b,c,d**) different perspectives of the point cloud retrieved from a round trip scanning path from both sides.

**Figure 11:**
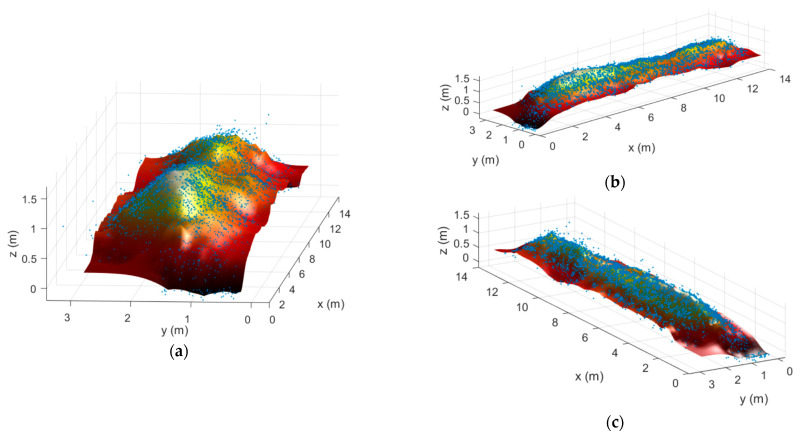
Surface reconstruction of the real stockpile from different perspectives.

Since both side scans can be referred to the GCS, the retrieved data can be merged and the point cloud is referred to the same visualization coordinate system. The rover modules positions at each measurement instant along with the retrieved point cloud are presented in Figure 10. For the sake of compactness, Figure 11 just shows the reconstruction results obtained when applying *proc2* from three different perspectives. From these results, the three different sections that constitute the stockpile can be clearly observed, concluding that a complete model of the stockpile can be retrieved using the proposed technique.

**Figure 12:**
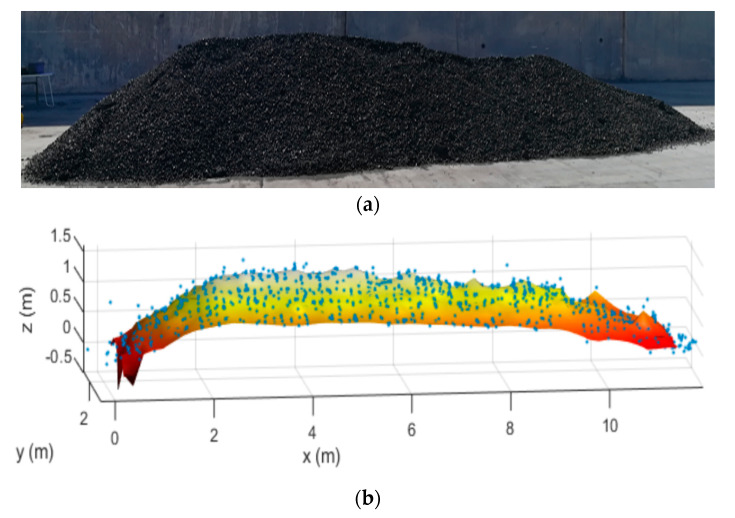
(**a**) Picture and (**b**) surface reconstruction of the stockpile.

In order to evaluate the robustness of the proposed system, the stockpile was modified, with the new one having a height and length of 1.5 m and 11.6 m, respectively (see Figure 12), and a new scan was conducted. In Figure 12b the results obtained after reconstructing the scanned data are shown. It can be seen that the reconstruction closely follows the changes of the stockpile geometry, confirming the robustness of the proposed system and the suitability of the applied methods.

**Figure 13:**
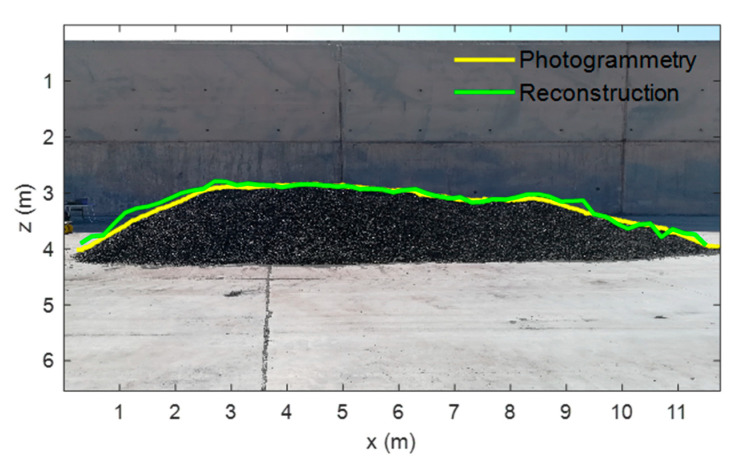
Picture of the stockpile, with the profile being recovered using a photogrammetry-like technique shown in yellow and the profile obtained from the reconstruction in green.

Aiming at assessing the accuracy of the proposed system, a photogrammetry-like technique is employed to compare the profiles of both the real and reconstructed stockpile. The true profile of the stockpile was extracted from a picture took during the measurements, which corresponds to the yellow line in Figure 13. This profile is compared with the one obtained after the reconstruction (green line in Figure 13). It can be noticed the correlation between both curves, being almost identical on the middle section and having slight deviations on the lateral sections, which can be attributed to other scattering sources, as the scanner arrives at the ends of the stockpile. 

Analyzing the previous results, it has been found that the mean absolute error on predicting the stockpile profile height is 6.4 cm and the root mean square deviation is 8.6 cm. These errors are mainly due to the sensors precision and resolution, as well as due to the employed calibration and positioning methods. However, it has been shown that the system provides high precision to retrieve the stockpile profile. Indeed, the agreement between the outline and shape of the real and reconstructed stockpiles is very good. Moreover, the errors are much smaller than the ones presented in other works [15,16,17,18,19,20,21,22,23,24,25,26,27,28,29,30,31,32,33,34,35], always being much less than 20 cm, which is an acceptable level [12]. 

#### Real-Time Reconstruction

**Figure 14:**
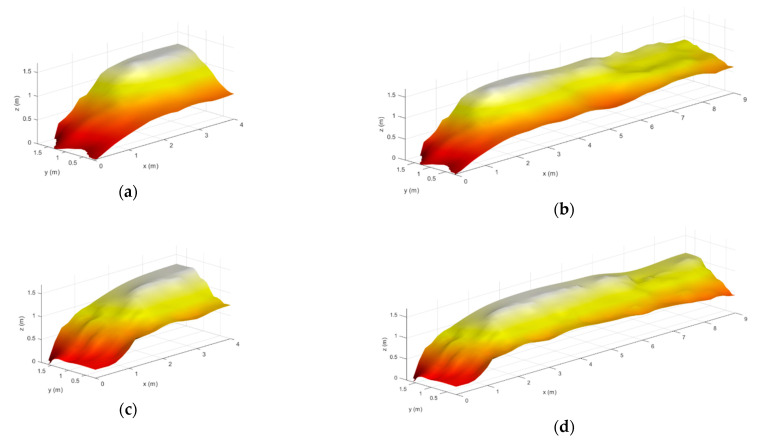
Reconstruction of the two real stockpiles when the scanner system is at (**a**,**c**) 4 m and (**b**,**d**) 9 m from the beginning of each stockpile.

In this section, a viability analysis for reconstructing the stockpile in real-time is conducted. Therefore, only the data retrieved until a given instantaneous measurement position is used for reconstructing the stockpile. As the radars have the capability of scanning the stockpile in three dimensions, the point cloud retrieved at each measurement instants contains points not only in the scanning plane (ZY plane shown in Figure 5a), but also from previous and forward stockpile parts. This allows us to redefine the reconstructed image with the information provided from successive measurement acquisitions, making the method adaptive in a certain way. The results of this real-time reconstruction are shown in Figure 14 for the two real stockpiles previously analyzed at two different instants. Consequently, it can be concluded that the proposed system and the employed methods are suitable for real-time scanning and reconstruction applications.

## 4. Discussion

The electromagnetic scanning system presented in this article gathers most of the requirements demanded by the Industry 4.0, regarding the connection between systems and the management of data. In this article, a new system to estimate the topography of stockpiles has been fully developed, from the selection and arrangement of their core components to the managing and processing of the acquired data. Moreover, the system does not rely on mathematical or geometrical models used in the literature to predict the initial shape of stockpiles, which cannot continuously monitor the structure, causing inefficient handling of the industrial processes [9,10,11].

A robust and highly accurate technique for managing the sensors data and reconstructing the stockpile topography from the obtained point cloud has been presented. It is worth noting that it avoids the usage of other models that cannot provide a continuous dynamic monitoring of the scanned structure and/or other time consuming and less reliable techniques that greatly depend on the selected algorithm [11]. 

The proposed system is cost-effective, mainly thanks to the use of commercial off-the-shelf components, whose prices are dropping due to their mass production. 

In contrast to other works that propose alternatives that are only assessed in laboratory conditions, the solution presented in this article has been tested in a realistic environment, obtaining highly precise reconstructions of the scanned structure.

It should be noticed that it is not easy to analyze the accuracy of the obtained results when scanning large structures, as the true shape and dimensions of such structures are not known. Indeed, there are not many works in the literature that quantitatively compare the obtained results from the scanning process with the actual structure. Nonetheless, in this article two techniques have been proposed to perform such comparisons. The first one relies on the measurements of the structure dimensions taken in the field, whereas the other one uses a photogrammetry-like technique to extract the profile of the scanned structure. From both methods, a high accuracy on the structure reconstruction has been clearly obtained. In fact, the proposed system performs better in terms of accuracy than other works proposed in the literature [15,35]. 

Finally, it should be noted that although the data retrieved from the sensors is downloaded after the scanning process, the viability of reconstructing the surface in real-time has been verified. 

## 5. Conclusions

In this article, a new and highly precise system has been presented for electromagnetically scanning large structures. The system combines the range information provided, as point clouds, by an array of mm-wave radars with the highly accuracy positioning data provided by GNSS-RTK modules, forming a sensor-fusion system that enables to merge the point clouds taken from different arbitrary positions. Moreover, communication and control components have been employed to send and receive data from the sensors and to manage the system status. A laptop has been also used for fusing the geo-referred data and properly reconstructing the scanned structure. Therefore, a new methodology to achieve 3D images of any large structure, object or group of objects, even when dust or heavy smoke are present and regardless of the ambient light, is introduced. As a proof of concept, the system has been tested on a stockpile-alike model and in a realistic environment at a seaport with a scaled coal stockpile, obtaining accurate results in both cases.

The reconstructed model has been compared with the true scanned structure dimensions, giving small errors (on the order of centimeters). For further verification of the system accuracy, a photogrammetry-like technique is used to compare the reconstructed profile with the true one, showing also small discrepancies. Therefore, the precision on the reconstruction of the structure has been validated, as well as the proper performance of the sensors that comprise the system.

In addition, the system and the proposed reconstruction method have been tested under real-time conditions, showing an excellent performance.

It should be noticed that although the proposed system is a proof-of-concept for scanning reasonably large structures, it can be easily scaled. Moreover, it can be used in other outdoor applications involving the retrieving of an electromagnetic image of large structures.

## Figures and Tables

**Table 1:** Real and reconstructed dimensions of the stockpile.

	Section 1		Section 2	
	Height (m)	Length (m)	Height (m)	Length (m)
Real dimensions	1.20	2	1.50	3.4
Reconstructed Stockpile	1.25	2	1.53	3.4

**Figure A1:**
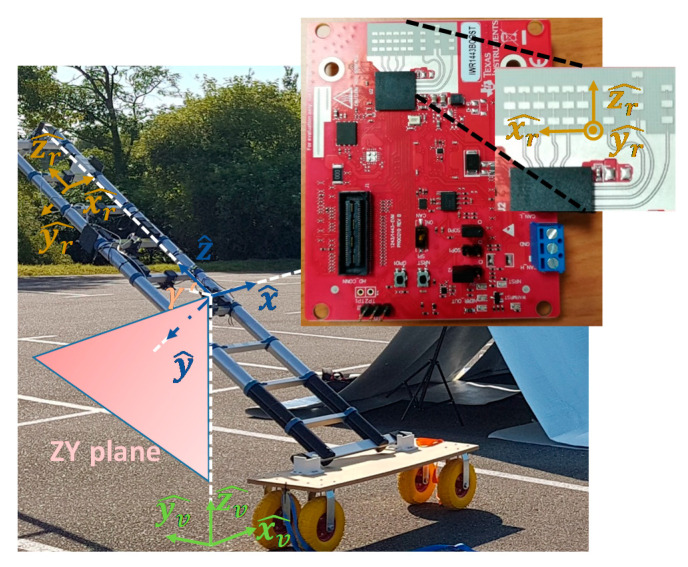
Coordinate system representation.

**Figure A2:**
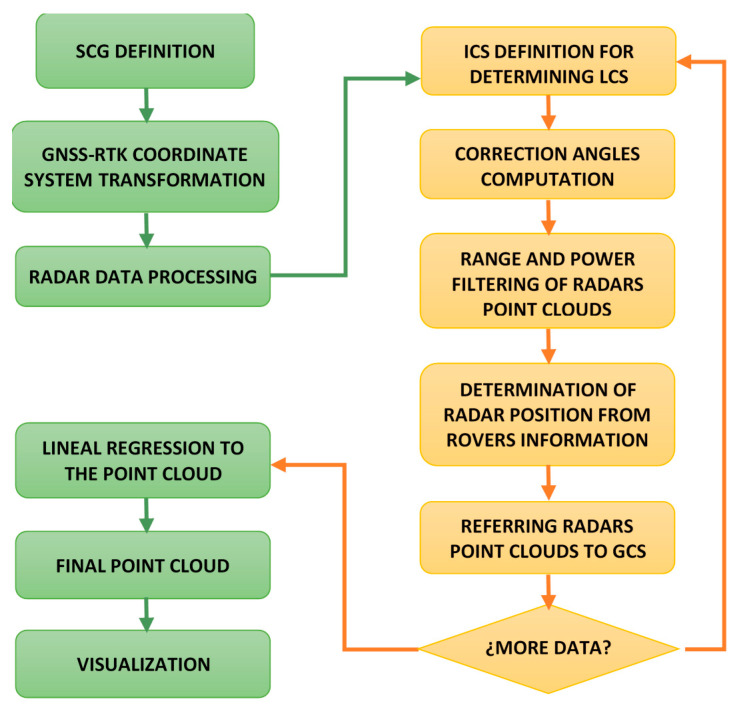
Data processing flow chart.

**Figure A3:**
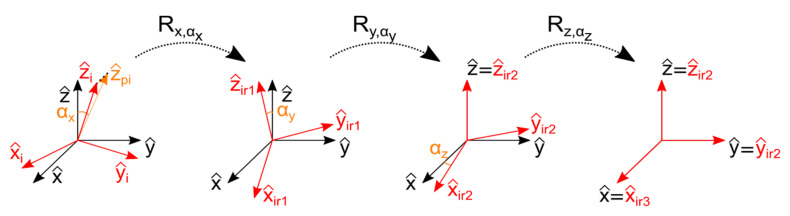
Coordinate system transformation to get the ICS vectors ($\hat{x_{i}}$,$\hat{y_{i}},\hat{z_{i}}$), and hence the LCS of each radar, equally oriented as the GCS ones ($\hat{x}$,$\hat{y},\hat{z}$).

## Data Availability

The data presented in this study are available in the article figures and tables.

## Appendix A

The point cloud provided by each radar on each measurement position is related to its own local coordinate system (LCS). Aiming at reconstructing the scanned surface, the aforementioned point clouds should be referred to a global common fixed coordinate system (GCS). This system is defined by three orthonormal vectors ($\hat{x},\hat{y},\hat{z})$, which are determined from the positioning data provided by the GNSS-RTK modules as follow:

- $\hat{x}$ vector → obtained by computing the mean of the retrieved positions provided by the rover modules along the longitudinal scanning of the stockpile (from the beginning to the end of the stockpile).
- $\hat{z}$ vector → defined by the vector connecting the two rover module positions.
- $\hat{y}$ vector → cross product between $\hat{x}$ and $\hat{z}$ vectors.

The advantages of using the aforementioned coordinate system is that their constituent vectors (blue triple ($\hat{x}$,$\hat{y},\hat{z})$ in Figure A1) are almost parallel to the local systems ones of the radars (brown triple ($\hat{x_{r}}$,$\hat{y_{r}},\hat{z_{r}}$) in Figure A1). Once the point clouds of the radars are defined with respect to the GCS, an additional transformation has to be conducted to refer them to the visualization coordinate system (green triple ($\hat{x_{v}}$,$\hat{y_{v}},\hat{z_{v}})$ in Figure A1). The latter provides a better representation of the structure, so that the $\hat{z_{v}}$ vector points to the sky, whereas the $\hat{x_{v}}$ and $\hat{y_{v}}$ vectors are parallel to the ground.

The positioning information is given by the rover modules on the ECEF (*Earth-centered, Earth-fixed*) coordinate system. Therefore, a coordinate system transformation is firstly required for referring this positioning information to the GCS.

Figure A2 shows the data processing flow chart followed in this article. Firstly, the global coordinate system is defined (as previously indicated) and the positioning data provided by the rover modules are referred to the GCS. Then, the data from the radars are processed for each measurement instant by following the steps described below (blocks in yellow in Figure A2):

- Computation of an instantaneous coordinate system (ICS), which is defined by the auxiliary vectors ($\hat{x_{i}}$,$\hat{y_{i}},\hat{z_{i}})$. These vectors are computed in a similar way as the GCS previously defined, except for $\hat{x_{i}}$ that is calculated from several consecutive measurement positions (four in this case). These ICS allows to determine the radars orientation on each measurement instance and, hence, its LCS.
- The correction angles that determine the orientation of the ICS (and hence the LCS) regarding the GCS (see Figure A3) are calculated as follows:
  ◦ $\alpha_{x}$ is extracted by projecting the vector $\hat{z_{i}}$ on the ZY plane (obtaining $\hat{z_{pi}}$) and computing the angle between $\hat{z_{pi}}$ and the XZ plane. After applying the rotation by an angle $\alpha_{x}$ around the $\hat{x}$ axis to the vectors ($\hat{x_{i}}$,$\hat{y_{i}},\;\hat{z_{i}})$, the triad ($\hat{x_{ir1}},\hat{y_{ir1}},\hat{z_{ir1}})$ is obtained.
  ◦ $\alpha_{y}$ will be the angle between the $\hat{z_{ir1}}$ and the YZ plane.
  ◦ $\alpha_{z}$ will be the rotation angle required to make the triad ($\hat{x_{ir1}},\hat{y_{ir1}},\hat{z_{ir1}})$ and ($\hat{x}$,$\hat{y},\hat{z})$ coincident.

Once the correction angles are extracted, the rotation matrices are applied to transform the ICS, so that its triad vectors ($\hat{x_{i}}$,$\hat{y_{i}},\hat{z_{i}})$ have the same orientation as the ones of the GCS ($\hat{x}$,$\hat{y},\hat{z})$.

- A filtering based on the range and reflected power is firstly applied to the point clouds provided by each radar at each measurement instant for discarding unwanted points. These points are detected with a low power or far away from the radars and in dispersed areas, so they cannot be attributable to the scanned structure.
- The translation matrix from the ICS of each radar to the GCS is determined using the relative positions of the radars regarding the rover modules defined by an initial calibration. Using both the translation matrices and the rotation ones, it is possible to refer the point clouds provided by the radars at each measurement instant in the GCS.

Once all the data is processed, a linear regression is applied to the final point cloud (which is defined regarding the GCS). For doing so, the points on the cloud that lies at a certain distance from the ZY plane (see Figure A1) are selected for computing a lineal regression, discarding the points that lie far away from this lineal regression. Although the latter filtering process is computed at each measurement instant, it can be conducted at any other interval.

Finally, an additional transformation is applied to represent the point cloud in the visualization coordinate system (green triad vectors ($\hat{x_{v}}$,$\hat{y_{v}},\hat{z_{v}})$ in Figure A1). The latter transformation is obtained by rotating the point cloud $\beta =90^{\circ }-\gamma$ degrees around the $\hat{x}$ vector, being γ the angle between $\hat{z}$ and the ground (obtained from the rover modules positioning information), and a translation regarding $\hat{z_{v}}$ (allowing to refer the point cloud with regards to the ground). This transformation is useful for a better interpretation of the retrieved point cloud.
